# Basis of apomixis in flowering plants – state of the art and research perspectives

**DOI:** 10.1007/s00497-026-00540-w

**Published:** 2026-05-30

**Authors:** Agnieszka Barbara Springer, Krystyna Musiał

**Affiliations:** 1 The Franciszek Górski Institute of Plant Physiology, Polish Academy of Sciences, Niezapominajek 21, Cracow, 30-239 Poland; 2 Department of Plant Cytology and Embryology, Institute of Botany, Jagiellonian University, Gronostajowa 9, Cracow, 30-387 Poland

**Keywords:** Apomeiosis, Clonal seeds, Mutants, Ovule, Plant breeding, Parthenogenesis

## Abstract

In most angiosperm plants, seeds are produced through a sexual process. Some angiosperms have developed an alternative reproductive strategy termed apomixis which involves producing seeds without the key steps of sexual development (i.e. meiosis, fertilization) and results in progeny genetically identical to the mother plant. Despite significant research advances, the evolutionary origin and molecular nature of apomixis remain unclear. Recent findings suggest that apomixis may have emerged from a sexual pathway deregulated by genetic and epigenetic modifications and stress signals (both endogenous and exogenous) that led to the alteration or omission of selected stages of sexual development. Apomixis, as a natural phenomenon that enables clonal reproduction by seeds, is a desirable trait with great potential in plant breeding, especially in terms of preserving hybrid vigor and gene combinations of elite phenotypes over subsequent generations. Notwithstanding, apomixis does not occur in major crops and therefore research programs focus on how to introgress, induce, or mimic apomixis in agronomically significant sexual species. The present review outlines the history of research on apomixis in flowering plants, its mechanisms, and a summary of the latest and most impactful research advances that may pave the way for the introduction of apomixis to sexually reproducing crops. The reader’s attention is also drawn to the partially explained issue of intercellular communication in ovules during early apomictic processes in the context of the cell wall’s changed chemical composition in cells that enter the apomictic developmental pathway.

## Key Message

The review summarizes what we already know about apomixis, starting from early research and ending with the latest reports that present the genetic basis of apomixis and outline future perspectives.

## Introduction

The term apomixis, introduced by Winkler (1908), originally had a very broad meaning and defined all types of asexual reproduction in which a new plant organism developed without gamete fusion, including various forms of vegetative propagation (Nogler 1984; Asker and Jerling 1992; Carman 1997). Despite the fact that phenomena similar to apomixis are also known in some ferns (Liu et al. 2012; Grusz 2016), the term apomixis is currently restricted to agamospermy, i.e. reproduction through asexually produced seed which results in clonal offspring identical to the maternal genotype (Nogler 1984; Asker and Jerling 1992; Bicknell and Koltunow 2004; Van Dijk 2009). In the past, apomictic lineages were considered to be evolutionary dead ends, but the current understanding of apomixis clearly highlights the significant role of apomictic reproduction in the diversification of polyploid complexes and the evolution of angiosperms; therefore, apomixis is assumed to actually promote immortality (Richards 2003; Hörandl and Hojsgaard 2012; Hojsgaard et al. 2014, 2019).

Asexual seed formation was first described in the dioecious species *Alchornea ilicifolia* (Euphorbiaceae) by Smith (1841) who observed that female plants derived from Australia and cultivated in Kew Gardens produced functional seeds despite the absence of pollen donor plants. Pioneer cyto-embryological studies of apomictic species were undertaken at the turn of the 20th century (reviewed in: Asker and Jerling 1992; Cornaro et al. 2023) and *Antennaria alpina* (Asteraceae) was the first examined apomict (Juel 1898, 1900). Research findings at that time clearly indicated the relationship between a high number of chromosomes, hybrid origin, and apomixis (Richards 1973). It was therefore concluded that interspecific hybridization and polyploidy could be important prerequisites for apomixis in addition to genetic background (Grimanelli et al. 2001a; Whitton et al. 2008). The 1940s saw rapid developments in the research on the cytological and genetic basis of apomixis (Stebbins 1941; Gustafsson 1946, 1947a, b; Brown and Emery 1958; Nogler 1984; Richards 1986; Bashaw and Hanna 1990). It was shown that apomixis is inherited as a dominant trait and the application of modern tools in molecular biology made it possible to identify several loci involved in the independent genetic control of apomixis components (Hand and Koltunow 2014 and references therein; Wang et al. 2017; van Dijk et al. 2020; Xu et al. 2021). Discoveries regarding the genetic basis, molecular aspects, mechanisms of epigenetic regulation of apomixis and the impact of other endogenous and exogenous factors have been summarised in a number of recent reviews (Barcaccia et al. 2020; Hojsgaard 2020; Schmidt 2020; Palumbo et al. 2022; Xu et al. 2022; Cornaro et al. 2023; Terzaroli et al. 2023; Goeckeritz et al. 2024; Heidemann et al. 2025; Hu et al. 2025). Numerous publications have also reviewed the recent advances in synthetic apomixis systems as a potential tool for clonal seed production in modern plant breeding (Fiaz et al. 2020; Ozias-Akins and Conner 2020; Scheben and Hojsgaard 2020; Wang 2020; Underwood and Mercier 2022; Yin et al. 2022; Li et al. 2023; Mahlandt et al. 2023; Vernet et al. 2023; Xiong et al. 2023).

For a complete understanding of the intricacies of research on apomictic seed formation, this article introduces the reader to the process of seed formation in angiosperms, starting with sexual species and then characterizing the types of apomictic reproduction. We present the current state of knowledge on the genetic and molecular basis of apomixis and outline contemporary trends in research.

## Sexual processes in angiosperm ovules

**Fig. 1:**
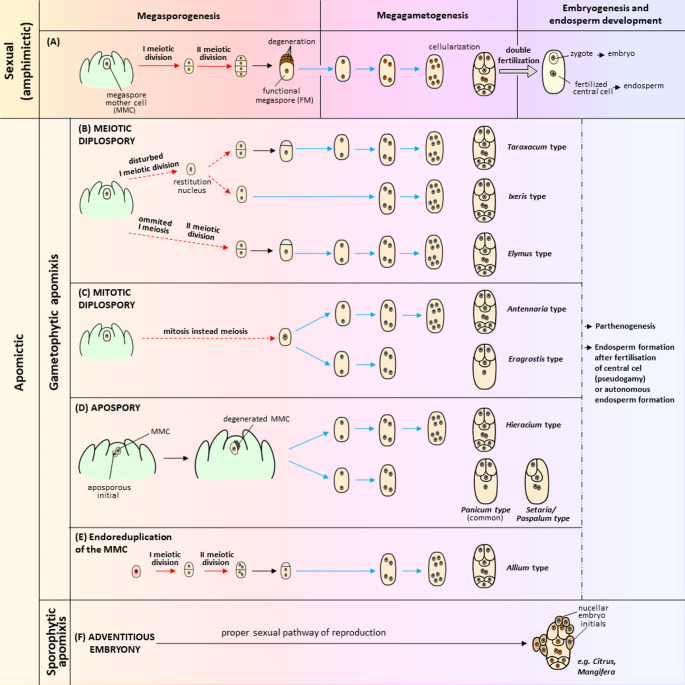
An overview of sexual reproduction (**A**) and types of apomixis (**B**-**F**). The main criteria for classifying types of apomixis relate to the course of meiotic division (disturbed, omitted, or replaced by mitosis), which cells undergo division, and the number of mitotic divisions leading to the formation of a mature female gametophyte. The red arrows indicate proper meiosis, while the arrows with a red dashed line indicate abnormal meiotic division; mitotic divisions are marked with blue arrows. The ploidy level of the cell nuclei is indicated by different colors – orange for haploid nuclei and gray for diploid nuclei. The cell nucleus undergoing endoreduplication is highlighted in red

Most angiosperms produce seeds by sexual reproduction (Fig. 1A). Their lifecycle alternates between haploid and diploid phases which are initiated by meiosis and gamete fusion, respectively. Both phases are represented by multicellular organisms, namely the diploid sporophyte generation (spore-producing organism) and the haploid gametophyte generation (gamete-producing organism). The female gametophyte (embryo sac) develops in the nucellus of the ovule and involves two steps, i.e. megasporogenesis and the subsequent megagametogenesis (Huang and Russell 1992; Reiser and Fischer 1993; Yadegari and Drews 2004). Given the variety of developmental patterns, the monosporic development of the *Polygonum*-type, which has been confirmed in over 70% of angiosperms (Willemse and van Went 1984; Huang and Russell 1992; Reiser and Fischer 1993; Yadegari and Drews 2004; Drews and Koltunow 2011), is the most common pattern of female gametophyte development described in flowering plants. In the ovule primordium of these flowering plants, a single archesporial cell differentiates which usually functions directly as a megaspore mother cell (Bouman 1984; Drews and Koltunow 2011). This diploid cell undergoes meiosis to produce four haploid megaspores three of which (typically those located closer to the micropylar pole of the ovule) undergo cell death and the chalazal cell becomes a functional megaspore. The transient deposition of callose in the cell wall of the megaspore mother and its subsequent deposition in the cell walls of the megaspores is an inherent feature of this particular type of megasporogenesis (Rodkiewicz 1970; Bouman 1984; Webb and Gunning 1990; Reiser and Fischer 1993). The understanding of callose’s role in megasporogenesis and the selection of functional megaspore remains limited. It is assumed, however, that callose may act as a molecular filter that selectively regulates the intercellular communication program at the switch from sporophytic to gametophytic phase (Bouman 1984; Drews and Koltunow 2011; Tucker and Koltunow 2009, 2014; Cornaro et al. 2023). In the course of megagametogenesis, the functional megaspore undergoes three mitotic divisions without cytokinesis, which results in the formation of an eight-nucleate embryo sac. At the final stage of megagametogenesis, cellularization occurs in the embryo sac and four distinct cell types differentiate that are appropriately arranged along the micropyle-chalazal axis. In addition to genetic factors involved in this process, the phytohormone auxin acts as a key signalling molecule for cell fate specification within the female gametophyte (Pagnussat et al. 2009; Sundaresan and Alandete-Saez 2010; Lituiev 2013; Tekleyohans et al. 2017; Skinner and Sundaresan 2018). The mature *Polygonum*-type embryo sac is a polarized seven-celled structure composed of highly specialized cell types, namely one egg cell and two synergid cells at the micropylar pole, a central cell that initially contains two haploid polar nuclei that later fuse to form a secondary nucleus, and three usually short-lived antipodal cells at the chalazal pole. Unlike antipodal cells, synergids are essential for reproduction since they control many steps of the double fertilization process, including pollen tube guidance, pollen tube growth arrest in the female gametophyte, pollen tube discharge and the migration of sperm cells (Punwani and Drews 2008 and references therein). During the fertilization process in angiosperms, two sperm cells are delivered by the pollen tube into one of the synergid cells, which degenerates after receiving the male gametes. Next, one of the sperm cells fuses with the egg cell and the other one with the central cell, which initiates the development of the embryo and endosperm (Bleckmann et al. 2014; Dresselhaus et al. 2016). The persistent synergid cell degenerates shortly after fertilization of the central cell, and its fertilization-dependent elimination is essential for blocking excess pollen tubes and thus preventing polyspermy (Maruyama et al. 2015; Chen et al. 2025).

## Apomictic pathways of seed formation

### Gametophytic apomixis

Depending on the origin of the apomictic lineage, two major types of apomixis are distinguished: gametophytic and sporophytic (also referred to as adventitious embryony) (Fig. 1B–F) (Nogler 1984; Asker and Jerling 1992). Gametophytic apomixis involves modifications to the developmental pathway of the female gametophyte. In the lifecycle of taxa with this mode of apomictic reproduction, alternation of generations is maintained, but the gametophyte and sporophyte have the same ploidy level as meiosis is omitted or altered to become non-reductional. The latter leads to an unreduced female gametophyte in which the apomeiotic egg cell develops parthenogenetically into a maternal embryo (Nogler 1984; Asker and Jerling 1992). The omission or deregulation of meiosis in gametophytic apomicts is often referred to as apomeiosis (Nogler 1984). The vast majority of gametophytic apomicts are polyploid, while *Boechera* (formerly *Arabis*), *Paspalum*,* Ranunculus* are some of the few known genera with apomixis at the diploid level in the wild (Asker and Jerling 1992; Naumova et al. 2001; Siena et al. 2008; Sharbel et al. 2009; Schinkel et al. 2016).

Gametophytic apomixis is subdivided into diplospory and apospory based on the origin of the cell that initiates the development of the unreduced female gametophyte. In diplospory, the female gametophyte develops from the megaspore mother cell via modified meiosis in which neither recombination nor reduction of chromosome number occurs, which leads to the formation of a dyad of unreduced megaspores (meiotic diplospory) (Fig. 1B) or by omitting meiosis and replaced it with mitotic division (mitotic diplospory) (Fig. 1C). Mitotic diplospory is more common and has a broader taxonomic distribution than meiotic diplospory which has been reported mainly in the apomicts of the Asteraceae family (Asker and Jerling 1992; Noyes 2007; Cornaro et al. 2023). In addition to numerous variations of mitotic and meiotic diplospory, one can distinguish the *Allium*-type diplospory (Fig. 1E) in which pre-meiotic endoreduplication occurs in the megaspore mother cell, followed by regular meiotic division and the formation of four unreduced nuclei (Håkansson and Levan 1957; Kojima and Nagato 1992). In most diplosporous apomicts, the mature female gametophyte is seven-celled, while its structure and organization resemble a meiotic embryo sac of the *Polygonum*-type (Leblanc et al. 1995; Bonilla and Quarin 1997; van Baarlen et al. 2002; Musiał and Kościńska-Pająk 2013; Janas et al. 2016).

In apospory, the unreduced female gametophyte develops from a somatic nucellar cell that acquires the developmental competence of a functional megaspore. One or more aposporous initial cells may differentiate in the vicinity of a meiotically dividing cell, but only a single aposporous initial typically undergoes mitotic divisions to form an unreduced female gametophyte when the sexual pathway is suppressed (Koltunow et al. 1998; Tucker et al. 2003); however, there are also cases of ovules in which several aposporous embryo sacs develop and seeds with multiple embryos may be formed (Kaushal et al. 2018; Carman et al. 2019). There are two types of aposporous embryo sacs (Fig. 1D). The arrangement of cells in embryo sac of *Hieracium*-type is bipolar and morphologically similar to the meiotic embryo sac of the *Polygonum*-type, while the *Panicum*-type (found in some grasses only) is a monopolar four-celled embryo sac with no antipodal cells (Warmke 1954; Koltunow 1993; Koltunow et al. 1998).

Regardless of how the unreduced female gametophyte forms, the apomeiotic egg cell can develop parthenogenetically, which leads to the formation of clonal progeny with the maternal genotype. As in sexual species, the formation of endosperm in gametophyte apomicts is crucial to ensure coordinated embryo growth. In diplosporous and aposporous species, the endosperm can develop spontaneously without fertilization of the central cell (autonomous apomixis) or it requires pollination and fertilization of the central cell to grow (pseudogamous apomixis or pseudogamy). In most apomictic plants (ca. 90%), fertilization of the central cell remains a prerequisite for endosperm development. Only in a few apomictic taxa, mainly from the Asteraceae family (e.g., *Erigeron*, *Hieracium*, *Pilosella*,* Taraxacum*) does the endosperm develop autonomously (Nogler 1984; Mogie 1992; Noyes et al. 2007; Ogawa et al. 2013; Hands et al. 2016; van Dijk et al. 2020).

Most apomicts, including many autonomous ones, produce either reduced or unreduced pollen. This fact implies that the genetic factors that control apomixis may be transmitted via male gametes, which may prove vital in the establishment and spread of apomixis (Mogie 1992; Asker and Jerling 1992; Bicknell and Koltunow 2004; Whitton et al. 2008; Hojsgaard and Hörandl 2019). However, pollen produced by apomictic plants often exhibits reduced viability and functionality, which limits its ability to efficiently transmit apomixis-related alleles (Whitton et al. 2008; Janas et al. 2016; Souza-Pérez et al. 2021). Nevertheless, pollen-mediated transmission of apomictic genes may contribute to the origin of new apomictic lineages (Hojsgaard and Hörandl 2019). Apomictic plants are mostly facultative apomicts that retain the ability to form seeds sexually, which results in genetic variation in the offspring and contributes to clonal diversity (Hörandl 2024; Hörandl et al. 2025).

### Sporophytic apomixis

In the case of sporophytic apomixis, one or more maternal embryos develop by mitosis directly from the somatic cells of the ovule (typically from nucellar cells and marginally from integument cells) in parallel to the common processes of sexual reproduction (Fig. 1F) (Asker and Jerling 1992; Koltunow 1993; Naumova 1993; Gupta et al. 1996). Due to the fact that further development and maturation of adventitious embryos depend on the presence of endosperm as the source of essential nutrients and growth signals, they subsequently enter the fertilized female gametophyte. Seeds resulting from sporophytic apomixis may contain multiple embryos of both maternal and zygotic genotype, but adventitious embryos often outcompete zygotic embryo during seed development (Koltunow 1993; Naumova 1993; Whitton et al. 2008; Xu et al. 2021). Taxonomically speaking, sporophytic apomixis is the most widespread form of agamospermy and occurs more frequently in diploid species, while gametophytic apomixis is strongly correlated with polyploidy (Asker and Jerling 1992; Richards 2003; Hojsgaard et al. 2014). Adventitious embryony is more common in tropical than in temperate flora and is mainly found in the members of the Rutaceae, Orchidaceae, Celastraceae, and Melastomataceae family (Naumova 1993; Whitton et al. 2008; Hojsgaard et al. 2014; Firetti 2018; Xu et al. 2021; Mahlandt et al. 2023). Adventitious embryony has been found to occur in some economically significant plants, e.g. in the species of the *Citrus*, *Mangifera*, *Garcinia*, *Ribes*, *Beta*, and some species belonging to Poaceae (Batygina 1991; Naumova 1993; Yao et al. 2007; Xu et al. 2022). Recently, sporophytic apomixis has been extensively studied at the molecular level in *Citrus*, a model system for nucellar embryony research (Xu et al. 2021 and references therein), as well as in *Mangifera indica* (Yadav et al. 2023; Ali et al. 2025).

## Taxonomic occurrence of apomixis

A relatively small number of angiosperm species are capable of producing seeds by apomixis; it is considered to occur in approximately 0.1% of flowering plant taxa (Mogie 1992; Whitton et al. 2008; Hojsgaard 2020). It is estimated that approximately 2.3% of angiosperm genera contain apomictic species and there are no fully apomictic genera among flowering plants (Hörandl 2024). According to the latest findings, apomixis has been recorded in 32 orders, 74 families, 299 genera, and over 400 species. Some discrepancies in the numbers quoted by various studies are mainly due to different taxonomic approaches (Hojsgaard and Pullaiah 2023; Hörandl 2024; Hörandl et al. 2025). It should be noted, however, that less than 10% of angiosperm species have so far been studied using cyto-embryological techniques and it cannot be ruled out that, the current list of apomictic taxa may be expanded to include new species as research progresses (Albertini et al. 2019). On the same note, apomictic reproduction has been reported in many unrelated families of monocots and eudicots (Hojsgaard et al. 2014; Barcaccia et al. 2020). There are also data that evidence the occurrence of apomictic processes in some representatives of early-diverging angiosperms (Rudall et al. 2008; Smissen et al. 2019). Thus, the distribution of the trait indicates a polyphyletic pattern of apomixis origin. The different modes of apomictic reproduction are taxonomically scattered among flowering plants and it seems that apomixis has most likely arisen independently many times throughout the evolution of angiosperms (Barcaccia et al. 2020; Hojsgaard and Pullaiah 2023; Hörandl et al. 2025). The three species-rich families, i.e. Asteraceae, Rosaceae and Poaceae, account for 75% of confirmed cases of plants with gametophytic apomixis, (together they account for only 10% of flowering plant species). In addition, the key mechanisms of apomixis are different among them, i.e. Asteraceae representatives are usually diplosporous, while apomictic Rosaceae and Poaceae are mostly aposporous (Bicknell and Koltunow 2004; Whitton et al. 2008; Hojsgaard and Pullaiah 2023). In contrast, sporophytic apomixis is notably prevalent in the Rutaceae, Celastraceae and Orchidaceae family (Naumova 1993; Carman 1997). It remains unclear, however, whether the clustering of apomixis in certain angiosperm families is due to their genetic or developmental predisposition.

## The importance of apomixis in plant breeding

Sexually reproducing (amphimictic) species pass new combinations of genes (alleles) associated with a specific trait to their offspring, which leads to the offspring’s phenotypic variation; however, the elite genotype and the corresponding phenotype is lost in subsequent generations due to genetic segregation. In nature, duplication of the desired genotype and phenotype of the mother plant, in addition to vegetative propagation, is feasible via apomictic reproduction; therefore, apomixis - as a natural process that enables clonal reproduction by seeds - is a highly desirable trait in plant breeding. Unfortunately, most apomicts are insignificant crops and have no relatives of agricultural importance (Barcaccia and Albertini 2013). Although apomixis is found in distant relatives of some modern crop plants, such as *Pennisetum squamulatum* (related to millet), *Elymus* sp. (related to wheat) or *Tripsacum* sp. (related to maize), human activities to domesticate these species, including multiple crosses between wild varieties and the selection of some genotypes for further cultivation, may have suppressed their natural ability to reproduce by apomixis (Hanna et al. 1996; Grimanelli et al. 2001b). The introduction of apomixis into sexually reproducing major crops can become extremely beneficial in economic and social terms (Koltunow et al. 1995; Grossniklaus et al. 1996; Spillane et al. 2004; Barcaccia and Albertini 2013). Agricultural production uses cultivars that are first-generation progeny (F1) between two genetically distant lines, due to the highly beneficial effect of heterosis (hybrid vigor) on crop yields. The obtained plant material cannot be reused as seed source for the next F2 generation, as the resulting offspring will exhibit significant variation due to genetic segregation (Koltunow et al. 1995). For this reason, apomixis has the potential to preserve the hybrid vigor of F1 plants, because allows to clone desired trait over many generations. Controlled use of apomixis can bring direct benefits, such as simplifying the seed production process and drastically reducing its cost for selected, agronomically important plant genotypes, in particular ones grown as hybrids (Koltunow et al. 1995). Engineered apomixis would allow fixing elite genotypes that determine valuable traits, including resistance to pathogens or unfavorable environmental conditions, which is highly desired in light of the ongoing global climate change (van Dijk et al. 2016; Hojsgaard 2020). In addition, complications associated with sexual reproduction, such as incompatibility barriers or complex pollination strategies, could be avoided. Apomixis technology and cloning by seeds would provide added value to vegetatively propagated plants (e.g., potato, cassava) by generating disease-free material that is easier for storage and transportation (Spillane et al. 2004). Moreover, introducing apomixis into crops would offer an opportunity to create genetically homogeneous populations by making a large pool of genetic material available to further improve useful plants and create varieties adapted to local environmental conditions (van Dijk et al. 2016). Please note that these are just some of the promising prospects. There also is a view that the potential economic and social benefits from the application of apomixis in plant breeding could spark the next “Green Revolution” and mark a new era in plant breeding and seed production (Barcaccia and Albertini 2013). A bibliometric analysis conducted by Palumbo et al. (2022) showed that more than 1571 scientific articles on apomixis in plants have been published in the last 40 years, 1195 of which were published after the year 2000. This indicates that many research centers around the world are conducting extensive and multifaceted studies on this mode of reproduction. Notwithstanding the fact that the scientific advances of the last decade have greatly elevated our comprehension of apomictic reproduction, the implementation of apomixis technology into plant breeding remains a challenge for plant biotechnology.

## Genetic and molecular basis of apomixis

Sexual and apomictic development represent closely related pathways and there are indications that they may share common molecular regulatory features (Tucker and Koltunow 2009). Despite spectacular progress in research by means of advanced molecular tools, the mechanisms that condition apomictic processes are yet to be fully explored. There is no single hypothesis that can explain all the empirical data related to apomixis, its occurrence, evolution and molecular nature (Hojsgaard and Pullaiah 2023). Accordingly, three hypotheses are adopted to explain the genetic basis and origin of apomixis as a complex evolutionary trait with multiple viable sources of origins: (i) apomixis is the consequence of developmental asynchronies (spatial and temporal deregulation of the expression of genes active in the sexual development pathway) that result from hybridization and/or polyploidization (Koltunow 1993; Grossniklaus et al. 1996; Grimanelli et al. 2001a), (ii) apomixis is a mutation-based anomaly that involves a simple or complex genetic locus carrying genes for apomeiosis, parthenogenesis, and fertilization-independent endosperm development (Richards 2003; Ozias-Akins and van Dijk 2007), (iii) apomixis is an ancient switch, polyphenic to sex, triggered through epigenetic regulations (Carman et al. 2011; Grimanelli 2012; Albertini et al. 2019; Mateo de Arias et al. 2020).

A number of different model systems have been developed for apomictic species to study apomixis in its native form. They include taxa from the genera *Brachiaria*, *Eragrostis*, *Panicum*, *Paspalum*, *Pennisetum*/*Cenchrus*, *Poa*, *Tripsacum* in monocotyledons and taxa from the genera *Taraxacum*, *Pilosella* (formerly *Hieracium* subgenus *Pilosella*), *Hypericum*, *Erigeron*, *Ranunculus*, and *Citrus* in dicotyledons (for details, see Bicknell and Koltunow 2004; Hand and Koltunow 2014; Carballo et al. 2021; Hojsgaard and Pullaiah 2023). Apomixis has been demonstrated to be a heritable trait in all species studied to date. Genetic analyses with apomicts as pollen donors in crosses with sexual individuals as maternal parents have revealed that apomixis is inherited as a dominant trait and that its components, i.e. apomeiosis, parthenogenesis, and autonomous endosperm development, can be uncoupled, which suggests that they are controlled by independent loci (reviewed in Grossniklaus et al. 2001; Ozias-Akins and van Dijk 2007; Barcaccia and Albertini 2013; Hojsgaard and Pullaiah 2023). The following examples of apomictic model plants discussed here show that apomixis is determined by at least two independent loci or by clusters of tightly linked loci. For instance, apomixis in *Erigeron annuus* is controlled by two unlinked dominant loci, one for diplospory (*D*) and the other one (*F*) for both parthenogenesis and autonomous endosperm development (Noyes and Rieseberg 2000; Noyes et al. 2007). Similarly, two independent loci have been identified in *Taraxacum officinale*, one for diplospory (*DIP*) and the other one for parthenogenesis (*PAR*) (van Dijk and Bakx-Schotman 2004; Underwood et al. 2022), while the genetic control of autonomous endosperm formation in apomictic dandelions remains obscure (Van Dijk et al. 2020). Recently, the *PARTHENOGENESIS* (*PAR*) gene was isolated from the apomictic dandelion *Taraxacum officinale* and was shown to encode a K2-2 zinc finger domain protein with an EAR (ethylene-responsive element-binding factor-associated amphiphilic repression) motif (Underwood et al. 2022). In apomictic *Pilosella*, three genetic components contribute to apomictic seed formation, i.e., the *LOSS OF APOMEIOSIS* (*LOA*) locus which stimulates the formation of aposporous initials and is required for sexual suppression, the *LOSS OF PARTHENOGENESIS* (*LOP*) locus which controls both parthenogenesis and autonomous endosperm development (Catanach et al. 2006; Koltunow et al. 2011), and a third *AutE* locus essential for fertilization-independent endosperm formation (Ogawa et al. 2013; Henderson et al. 2017). In the genus *Boechera*, in which apomixis is expressed at the diploid level, the *APOmixis Linked LOcus* (*APOLLO*) is a genetic factor that may be associated with female apomeiosis (Corral et al. 2013), and its male counterpart is the *Unreduced Pollen GRAin Development2* (*UPGRADE2*) locus (Mau et al. 2013). The *UPGRADE2* is exclusively expressed in apomictic species, while the *APOLLO* shows different expression in apomictic and sexual ovules. The *APOLLO* has “sexalleles” and “apoalleles”, and the latter are characterized by a set of linked apomixis-specific polymorphism (Corral et al. 2013).

In apomicts from the Paniceae tribe within the Poaceae family, apomeiosis and parthenogenesis are inherited together as a single Mendelian locus (Hojsgaard and Pullaiah 2023 and references therein). For example, apomixis segregates as a single dominant locus in aposporous *Cenchrus squamulatus* (syn. *Pennisetum squamulatum*) (Conner et al. 2015; Ozias-Akins and Conner 2020). This locus is referred to as the apospory specific genomic region (ASGR), i.e. a physically large, hemizygous, non-recombining chromosomal region that contains multiple copies of the *PsASGR-BABY BOOM-like* (*PsASGR-BBML*) gene (Ozias-Akins et al. 1998; Roche et al. 1999; Conner et al. 2015). Ectopic expression of the *BBM* or *BBM-like* genes in sexually reproducing *Arabidopsis* and *Brassica* plants led to spontaneous formation of somatic embryos and cotyledon-like structures on seedlings (Boutilier et al. 2002). Further studies have shown that ectopic expression of *BBM* in the egg cells in *Brassica napus* and *Solanum lycopersicon* (Chen et al. 2022) as well as *Oryza sativa* (Masuo et al. 2025) induced embryo development without fertilization. As with *Cenchrus*/*Pennisetum* species, in the genus *Paspalum* – another archetypal model system for studying apomixis-related genes in grasses – the apomixis trait is associated with a single non-recombining hemizygous genomic region, the Apomixis Controlling Region (ACR) (Siena et al. 2014; Galla et al. 2019). The ACR could carry silencers of sexuality and enhancers of apomictic seed formation (Siena et al. 2016). For example, one of the genes physically linked to ACR in *Paspalum notatum* is *PnTgs1-like*, whose expression during reproductive development was significantly higher in nucellar cells of sexual plants compared to aposporous plants, and whose down-regulated expression correlated with initiation of the apomictic pathway (Siena et al. 2014). Furthermore, molecular characterization of *PnTgs1-like* revealed that this candidate gene encodes a trimethylguanosine synthase-like protein, whose function in mammals and yeast is essential for development and reproduction (Siena et al. 2014). In *Paspalum simplex*, it has been shown that an apomixis-linked sequence is homologous to subunit 3 of the ORIGIN RECOGNITION COMPLEX (ORC3), and it has been hypothesized that the *PsORC3a* gene, specific for apomictic genotypes, is associated with down-regulation expression of its functional homolog and with the development of an apomictic endosperm that deviates from the canonical genome ratio of 2(maternal):1 (paternal) (Siena et al. 2016).

Numerous pieces of experimental evidence indicate that apomixis loci are usually hemizygous and demonstrate a number of similarities to the Y chromosome of dioecious plants, including repression of recombination, accumulation of transposable elements and degeneration of genes (Ozias-Akins et al. 2003; Pupilli and Barcaccia 2012; Wang et al. 2012). Furthermore, the inheritance of sporophytic apomixis in the genus *Citrus* is associated with the transfer of a single dominant locus and the *CitRWP* gene was identified as the candidate gene responsible for nucellar polyembryony in these plants (Nakano et al. 2008, 2012; Wang et al. 2017). The candidate gene *CitRWP* contained a Miniature Inverted-repeat Transposable Element (MITE) inserted into its promoter region and it was assumed that this insertion might have been responsible for establishing embryogenic development in nucellar cells (Wang et al. 2017; Xu et al. 2021). Recently, a DNA methylome analysis revealed hypermethylation of MITE in the proximal region of the *CitRWP* gene in polyembryonic *Citrus*. In parallel, hypomethylation was found in the promoter of *CitRWP* without a MITE insertion in monoembryonic *Citrus* (Jia et al. 2021). These findings suggest that the activation of *CitRWP* gene expression in polyembryonic *Citrus* is linked to the hypermethylation of DNA in the region of MITE insertion. A similar mechanism underlying polyembryony has also been described in *Mangifera indica* (Yadav et al. 2023; Ali et al. 2025). The *MiRWP* gene, characterized by a specific insertion in the gene’s promoter region, has been identified as responsible for polyembryony in mango and as an ortholog of the *CitRWP* gene (Yadav et al. 2023). It should be noted that MITE transposon insertion has also been reported in the upstream region of the parthenogenesis gene in the apomictic dandelion (*Taraxacum officinale*) and hawkweed (*Pilosella piloselloides*), which suggests a case of parallel evolution (Underwood et al. 2022).

There presently is a growing body of evidence to suggest that other interrelated regulatory control mechanisms, in addition to epigenetic regulatory pathways, are also of importance. These include cell cycle control, regulation of protein turnover, signal transduction pathways and phytohormonal regulation (for details, see Schmidt 2020; Terzaroli et al. 2023).

Recently, through synteny analysis, the *APO* locus was mapped onto the *Eragrostis curvula* reference genome, and two genes that could be part of molecular pathways involved in apomeiosis were identified (Gallardo et al. 2025). This study showed that the sequence of the SNPs linked to apomeiosis exhibits homology to an E3 ubiquitin ligase, a RING-type protein involved in ubiquitin-dependent protein degradation via the 26 S proteasome. This pathway plays a key role in many cellular and physiological processes in plants, such as phytohormone signal transduction, genetic recombination and cell cycle progression (Chen and Hellmann 2013). Increased expression of homologous gene associated with the ubiquitin-proteasome pathway was also observed in the aposporous initials and aposporous embryo sacs in *Hieracium praelatum* (Okada et al. 2013). Differential activity of E3 ligases was also found in nucellar cells of sexual and apomictic *Boechera* accessions, indicating their potential importance in regulating germline development (Zühl et al. 2019).

Lastly, research findings also suggest a potential impact of the relationship between different environmental stress conditions and the relative response of plants to sex/apomictic determination. Abiotic factors that increase oxidative stress, such as prolonged photoperiod, drought, heat, cold, starvation, salinity and osmotic stress, increase the rate of transition from apomeiosis to meiosis in facultative apomictic plants (Gounaris et al. 1991; Hörandl and Hadacek 2013; Klatt et al. 2016; Rodrigo et al. 2017; Mateo de Arias et al. 2020; Selva et al. 2020). Thus, oxidative stress mediates alterations of developmental pathways and shifts in redox homeostasis may act as functional triggers for sexual development (Hörandl and Hadacek 2013).

## Contemporary trends in apomixis research

To date, the strategy of introducing apomixis into crops through the introgression of apomictic traits from wild relatives has been successful to a limited extent. Synthetic apomixis leveraging biotechnology to modify sexual processes so that they mimic the apomictic mode of reproduction is an alternative approach (Xiong et al. 2023). The following three steps are crucial for synthetic apomixis to succeed: (i) apomeiosis induction (modification of meiosis into a mitotic-like division), (ii) uniparental embryo formation (parthenogenesis or post-fertilization elimination of the maternal or paternal genome), (iii) formation of viable endosperm (Ozias-Akins and Conner 2020). Synthetic apomixis can make effective use of gene editing technologies, notably the Clustered Regularly Interspaced Short Palindromic Repeats (CRISPR)/CRISPR-associated protein (cas) strategy (Scheben and Hojsgaard 2020; Qi et al. 2023). For a detailed list of the many apomixis-related genes employed in synthetic apomeiosis and parthenogenesis, see Mahlandt et al. (2023) as an example. The induction of apomeiosis by mutation of the *DYAD/SWITCH1* gene in *Arabidopsis* (Ravi et al. 2008), the mutation of the homologous *AMEIOTIC1* (*AM1*) gene in *Zea mays* (Pawlowski et al. 2009) and particularly the creation of the *MiMe* (*Mitosis instead of Meiosis*) genotype in *Arabidopsis thaliana* (d’Erfurth et al. 2009) were pivotal steps towards engineering apomixis.

The *MiMe* system in *Arabidopsis*, which has been applied to produce unreduced and unrecombined clonal male and female gametes, combines mutations of three genes, i.e., *Atspo11-1* (prevents DNA double-strand break formation, and thus eliminates recombination and pairing), *Atrec8* (modifies chromatid segregation) and *Atosd1* (omits the second meiotic division) (d’Erfurth et al. 2009). Initially, it seemed that implementation of the *MiMe* technology in phylogenetically distant dicotyledons (*Arabidopsis*) and monocotyledons (such as cereals) would be a challenge, however it soon proved possible. The *MiMe* system has been developed in rice (*Oryza sativa*) via mutations of *OsREC8*,* OsOSD1* and *OsPAIR1* (an alternative to *SPO11-1* which is essential for recombination) (Mieulet et al. 2016). Recently, the *MiMe* system has been utilized to generate unreduced, clonal gametes in three hybrid genotypes of a tomato (*Solanum lycopersicum*). In this specific *MiMe* system, mutations of *SlSPO11-1*, *SlREC8*, and *SlTAM* (*SLTARDY ASYNCHRONOUS MEIOSIS*) were combined; and through the hybridization of *MiMe* hybrids it is possible to obtain tetraploid offspring maintained genome-wide heterozygosity (Wang et al. 2024). The next step towards obtaining clonal offspring is to either skip fertilization or eliminate the paternal genome in the fertilized egg. Genome elimination can be induced by manipulating the centromere-specific histone CENH3 (Ravi and Chan 2010). Moreover, a mutation in *MATRILINEAL* (*MTL* or *MATL)/NOT LIKE DAD (NLD)* gene encodes a pollen-specific patatin-like phospholipase A can trigger haploid induction in *Zea mays* (Gilles et al. 2017; Kelliher et al. 2017). *MATL* mutants have also been generated in rice and wheat (Yao et al. 2018; Liu et al. 2020). *MiMe* and *dyad* plants as female have been crossed with *GEM* (Genome Elimination induced by a Mix of CENH3 variants) lines in *Arabidopsis* to produce clonal offspring (Marimuthu et al. 2011). Furthermore, apomixis has been successfully introduced into hybrid rice by simultaneous editing of *OsPAIR1*, *OsREC8*, *OsOSD1* and *OsMTL* through a CRISPR/Cas9 system; the resulting quadruple mutant was named as *Fix* (*Fixation of hybrids*) (Wang et al. 2019). Similarly, the quadruple mutant *AOP* (*Apomictic Offspring Producer*) created by mutating *OsSPO11-1*, *OsREC8*, *OsOSD1*, and *OsMATL* was able to produce apomictic offspring (Xie et al. 2019). Hybrid clonal seeds have also been obtained in rice by combining *MiMe* mutant with the expression of *BBM1* (*BABY BOOM1*) in the egg cell (Khanday et al. 2019). Recently, the dandelion *PAR* gene has been shown to have the potential for asexual plant breeding by means of CRISPR/endonuclease9 (Cas9)-mediated mutagenesis (Underwood et al. 2022; Huang et al. 2024). Heterologous expression of this gene in the egg cells of sexual crop lettuce (*Lactuca sativa*) can trigger their division without fertilization and development of haploid embryo-like structures (Underwood et al. 2022). Likewise, *PAR*-induced parthenogenesis has been recorded in the monocot crop foxtail millet (*Setaria italica*) (Huang et al. 2024). Also, the *PsASGR-BBML* transgene, derived from a wild apomictic grass *Pennisetum squamulatum*, can induce parthenogenesis in sexual pearl millet, rice, maize and tobacco, leading to the formation of haploid plants (Conner et al. 2015, 2017; Zhang et al. 2020). Recent reports on cowpea (*Vigna unguiculata*) also indicate that the *VuBBML1* gene plays a functional role in embryo and endosperm formation. Ectopic expression of the *VuBBML1* gene in the egg cell and central cell, together with central cell fertilization following self-pollination, resulted in the production of viable seeds and subsequently haploid plants (Amasende-Morales et al. 2026, preprint).

The above-mentioned engineering approaches may hence be a promising alternative for programming haploids to rapidly obtain homozygous lines in the very first generation after chromosome doubling. The frequency of inducing clonal reproduction in crops often remains too low, which limits the application of synthetic apomixis in the field (Goeckeritz et al. 2024 and references therein). Nevertheless, in the case of rice, almost complete penetration of synthetic apomixis has been achieved – the generated hybrid plants produced over 95% clonal seed across multiple generations (Vernet et al. 2022). Further research should focus on identifying factors that can substantially improve the efficiency of this bioengineering strategy, and study of natural apomictic species may prove helpful in this regard.

The final step in synthetic apomixis, i.e. the formation of a viable endosperm, is yet another element that affects the efficiency of the apomixis engineering system. Genome dosage and imprinting are known to play a key role in endosperm formation (Baroux et al. 2002). In most sexual angiosperms, a 2:1 maternal-to-paternal genome ratio is crucial for this tissue to develop properly, while shifts in the ratio usually result in seed abnormalities (Scott et al. 1998; Tiwari et al. 2010; Lu et al. 2012). On the contrary, natural apomicts tolerate imbalances in genome dosage and develop viable seeds regardless of the genome ratio (Quarin 1999; Bellucci et al. 2023). This can, however, hamper the development of viable seeds in synthetic apomicts; therefore, bypassing imprinting and/or dosage barriers in the endosperm will facilitate the engineering of apomixis technology. Since sperm cell activation and fertilization of the central cell depends on EC1 protein secreted exclusively by the egg cell (Sprunck et al. 2012), the initiation of parthenogenesis may additionally block the fertilization of the central cell due to EC1 proteins deficiency (Vernet et al. 2022). Skipping the fertilization of the central cell is not a prerequisite for the induction of clonal seeds, but autonomous endosperm can also be induced artificially in sexual species. The *FIS-PRC2* (*FERTILIZATION-INDEPENDENT SEED-POLYCOMB REPRESSIVE COMPLEX 2*) genes and the phytohormone auxin play an important regulatory role in seed development upon fertilization (Figueiredo et al. 2015). *FIS-PRC2* complex may prevent the initiation of endosperm formation by silencing the *TAR1* (*TRYPTOPHAN AMINOTRANSFERASE OF ARABIDOPSIS1*) and *YUC10* (*YUCCA10*) genes which hold key functions in auxin biosynthesis and whose paternal expression could promote the initiation of endosperm development (Figueiredo et al. 2015). In *Arabidopsis*, mutations in *FIS* genes (*medea*, *fie*, *fis*, and *msi1*) result in fertilization-independent endosperm initiation even though endosperm does not undergo cellularization and the seed eventually aborts its development (Chaudhury et al. 1997; Grossniklaus et al. 1998; Köhler et al. 2003). It was found that the development of autonomous seeds in *fis* mutants co-occurs with the derepression of the maternal alleles of the paternally expressed *YUC10* gene which is normally expressed only in the endosperm after fertilization (Figueiredo et al. 2015; Figueiredo and Köhler 2018). *Fis* mutants concurrently exhibit ectopic activation of auxin reporters, which indicates fertilization-independent activation of auxin signalling (Figueiredo et al. 2015; Figueiredo and Köhler 2018). It has recently been shown that in *Oryza sativa*, the genetic and epigenetic regulation of endosperm development is analogous to that described in *Arabidopsis* (Tonosaki et al. 2021). Two *EMBRYONIC FLOWER2 (EMF2)* gene homologs have been identified: *OsEMF2a* and *OsEMF2b*, which encode zinc-finger containing components of PRC2 complex (Luo et al. 2009). A mutation of *OsEMF2a* caused the development of endosperm without fertilization, involving proliferation of the central cell nuclei with distinct cytoplasmic domains and accumulation of starch granules and protein storage vacuole-like structures (Tonosaki et al. 2021). The role of PRC2 in the regulation of endosperm development in rice is also confirmed by the results of the analysis of *PRC2 FERTILIZATION INDEPENDENT ENDOSPERM* (*FIE*) homologs (Wu et al. 2023). *Osfie1* and *Osfie2* double mutants showed asexual embryo development and autonomous endosperm formation at a high frequency, while ovules of single *Osfie2* mutants exhibited asexual pre-embryo-like structures at a lower frequency without fertilization. These results suggest that an autonomous endosperm facilitates asexual embryo development (Wu et al. 2023).

It should be noted that basic research in the field of apomixis continues by involving non-model species and relying on classical research methods in combination with modern molecular techniques. Apomictic reproduction changes a cell’s developmental program along with its metabolism which is subject to regulatory mechanisms. In addition to direct intercellular communication and hormone signaling, epigenetic regulation, including the activation of specific transcription factors, signaling with non-coding RNA molecules (miRNA, siRNA), modulation of gene expression and changes in protein conformation, is of particular importance to these mechanisms (Scheben and Hojsgaard 2020). The topic of communication between the ovule’s somatic cells and the cells that enter the sexual or apomictic development pathway, especially in the context of cell wall modification, is one of the lines of current research (Leszczuk and Szczuka 2018; Janas et al. 2022, 2023). Changes in the cell wall’s chemical composition are one of a cell’s responses to stress, callose being the transient component of the cell wall whose deposition is promoted by reactive oxygen species (ROS) (Luna et al. 2011). This polysaccharide plays an important regulatory role in intercellular signalling throughout a variety of biological processes, including early sexual and apomictic reproductive events (Rodkiewicz 1970; Tucker and Koltunow 2014; Juranić et al. 2018; Cornaro et al. 2023). Recent data indicate that callose is deposited around megasporocytes in sexual reproduction, potentially in response to ROS. By contrast, callose deposition does not occur or is incomplete in the walls of cells that initiate apospory or diplospory (Carman et al. 1991; Peel et al. 1997; Tucker et al. 2001; Musiał et al. 2015; Janas et al. 2022). High activity of polyamine biosynthesis and spermidine metabolism in these cells allows for the quenching of ROS, and thus the altered pattern of callose deposition (Schmidt et al. 2014). This may indirectly indicate a role of callose in regulating intercellular signaling. In *Citrus* the differentiated initial cells of the nucellar embryos are isolated from the surrounding nucellar cells by a thickened cell walls, and such modification is usually associated with stress response (Long et al. 2016). A comparative analysis of polyembryonic and monoembryonic ovules revealed that the callose synthase gene *UGT* and other cell wall modification-related genes (*LAC*, *ACHI* and *CHI*) – classified as “response to stress” category – were up-regulated in polyembryonic ovules (Long et al. 2016). Reports also indicate that arabionogalactan proteins (AGPs) are involved in cell-to-cell signal transduction in the ovules during early sexual and apomictic reproductive processes (Acosta-García and Vielle-Calzada 2004; Coimbra et al. 2007; Demesa-Arévalo and Vielle-Calzada 2013; Juranić et al. 2018; Leszczuk and Szczuka 2018; Janas et al. 2023). AGPs detected by JIM13 antibody have been shown to be early cellular markers of both sexual and apomictic cell lineages in aposporous *Pilosella* spp. (Juranić et al. 2018), facultative apomict *Fragaria* x *ananassa* (Leszczuk and Szczuka 2018) and diplosporous *Taraxacum belorussicum* (Janas et al. 2023). However, that reports regarding the role of AGP as a potential signal source in embryological processes still remains speculative and the mechanism of action has not been clarified. In spite of the enormous scientific efforts undertaken to date, further molecular studies are required to learn the principles of ovule-based intercellular signaling pathways in the early apomictic processes.

## Challenges and the questions that remain open

The findings outlined in this review represent merely a sample of the many advances in apomixis engineering (technology) that combine the approaches of genomics, transcriptomics and proteomics. Growing availability of genomic data and the continuous enhancement of molecular biology tools, especially with the use of intensively developed genome editing techniques, the practical application of synthetic apomixis in other crop plants is becoming increasingly feasible. Nevertheless, significant limitations remain, as each species is a unique case – taxonomic relationships and the evolution of ploidy must be taken into account, and it is therefore necessary to examine various mutation configurations (Heidemann et al. 2025). For example, it appears that the *MiMe* system cannot be used as a universal tool that would work in the same way for every species studied; all evidence suggests that it must be tailored to each specific case. An attempt to implement this mutation system in *Gossypium hirsutum* revealed severe limitations – the resulting transgenic plants were male-sterile and, even after pollination with wild type pollen, were unable to produce seeds (Qian et al. 2024). Also in the case of soybean (*Glycine max*) the implementation of the *Mime* system encountered limitations (Wang et al. 2025). Abnormalities in male meiosis (loss of chromosomes during the first meiotic division), low efficiency of skipping the second meiotic division have been reported. As a result, aneutetraploids were obtained instead of the expected tetraploids.

Thus, the question of what factors determine the successful transfer of the *MiMe* genotype to a variety of crops still remains unanswered. Numerous studies indicate that the successful implementation of genes responsible for the production of unreduced gametes is only a partial success; the problem is that clonal seeds formation has a low degree of expression, which poses a serious limitation (Scheben and Hojsgaard 2020; Heidemann et al. 2025). The performance of synthetic apomictic lines over multiple generations may be limited by unforeseen consequences, such as loss of genetic diversity and accumulation of deleterious mutations over time, thus a further challenge is to develop strategies to maintain genetic diversity within breeding populations. It should be emphasized that the system enabling synthetic apomixis is pseudogamy-like, which means that seed production still depends on the contribution of male gametes, which are essential for endosperm formation. A fully autonomous system allowing to produce clonal seeds without fertilization of the central cell has not yet been developed (Schmidt 2025). The absence of pollen in apomictic crop plants would be highly beneficial, as it ensures that apomeiotic egg cell will not be fertilized and it also eliminates the risk of introgression into sexual crop plants (Goeckeritz et al. 2024). In this regard as well, natural apomicts with autonomous seed formation can be a good source of new data. In the study of the molecular basis of sexual and apomictic processes, the location of female reproductive cells within the sporophytic tissues of the ovule appears to be important. A thorough understanding of the complex genetic and hormonal networks that regulate a cross talk between somatic and gametophytic cells may help to unravel the enigma of autonomous seeds formation. There is also the question of why only a few apomictic species (mainly from the Asteraceae family) are able to produce seeds spontaneously without fertilization? To clarify this issue, in-depth taxonomic, phylogenetic/phylogenomic analyses are necessary. Current research on the evolution of apomictic lineages places great emphasis on integrating data from the fields of morphology, cyto-embryology, ecology, and genetics, as combining these approaches can help solve the puzzle of the short- and long-term evolution of apomicts (Hörandl et al. 2025). Furthermore, advances in bioinformatics technology provide new tools for integrating the vast amounts of genomic data necessary to understand plant speciation and subsequent macroevolution (Karbstein et al. 2022). AI-based tools can be particularly helpful in the case of taxonomically complex groups of plants (Hörandl et al. 2025).

An important issue that should be taken into account is the legal framework governing the use of genetically engineered apomictic crops. Assuming a very optimistic scenario in which we have apomictic crop varieties (such as corn, rice, wheat, soybean) with complete penetrance of the apomixis trait in the population and high fertility, the implementation of apomixis technology in agriculture must comply with local legal regulations. Many countries may have significant restrictions on the use of this technology. Public perception may also play a role, as is the case with genetically modified organisms, which have always caused public controversy. In this respect, access to reliable information and effective education will certainly prove to be the solution here.

## Data Availability

No datasets were generated or analysed during the current study.
